# Influence of different treatment conditions on the filtration performance of conventional electret melt blown non-woven and novel nano FFP2 masks

**DOI:** 10.1371/journal.pone.0291679

**Published:** 2023-09-21

**Authors:** Robin Halamicek, Carolin Wiesmann, Richard Kröner, Matthias Eber, Christian Bogdan, Dirk W. Schubert

**Affiliations:** 1 Department of Material Science, Institute of Polymer Materials, Friedrich-Alexander-Universität Erlangen-Nürnberg, Erlangen, Bavaria, Germany; 2 Fiatec–Filter- und Aerosoltechnologie GmbH, Mainleus, Bavaria, Germany; 3 Mikrobiologisches Institut–Klinische Mikrobiologie, Immunologie und Hygiene, Universitätsklinikum Erlangen and Friedrich-Alexander-Universität Erlangen-Nürnberg, Erlangen, Bavaria, Germany; University of Sharjah, UNITED ARAB EMIRATES

## Abstract

To allow an efficient protection against viruses like the severe acute respiratory syndrome coronavirus type 2 (SARS-CoV-2), it is important to avoid their spreading by using filtering face pieces (FFP), which are categorized by different standards according to their filtration efficiency. In this study, we subjected six brands of FFP2 standard masks to three different conditions and subsequently analysed them for their filtration performance to evaluate potentials for reusability. The conditions comprised changes of temperature and air humidity, an exposure to isopropyl alcohol (IPA) and an autoclave sterilization. While four of six masks consisted of electrostatically treated melt blown non-wovens, two masks were fabricated using a nanofibrous multilayer system. Due to the absence of prior electrostatic treatment, the nano-masks did not show a significant change in filtration efficiency when discharged by IPA, unlike the melt blown nonwoven masks showing a significant decrease of filtration efficiency down to around 50% at a particle size of 0.3 μm. However, most melt blown masks maintained a sufficient filtration efficiency after all other treatments with even better results than the nanofibrous masks. This was particularly the case for the capacity to filter smallest particles/droplets with a size of around 0.1 μm, which is below the range of typical filtering standards and important for the retention of virally contaminated nano-aerosols or unattached viruses. After temperature/humidity variation and autoclave sterilization, melt blown masks were able to retain a filtration efficiency up to over 90% at 0.1 μm contrary to nano-masks showing a decrease down to around 70%. Based on their better filtration performance, lower price and potential reusability, we conclude that electret melt blown masks are the preferable type of FFP2 masks.

## Introduction

Due to the emergence of the SARS-CoV-2 pandemic and other respiratory infections, the demand for protective face masks has increased massively during the past 3 years [1]. Protective face masks can be classified by the efficiency in the filtration of particles with a defined size in the submicron scale. Established standards for FFP2 masks are defined by EN 149:2001 and N95 in NOISH 42 CFR Part 84, with comparable required filtration efficiencies of 94% at a particle size of ≥ 0.6 μm and 95% at ≥ 0.3 μm respectively. While these masks promise a personal protection against inhalation of virally contaminated aerosol droplets [2], they also hinder the active spreading of such droplets by acting as a mechanical barrier [3]. The filtration efficiency is strongly related to the fiber diameter of the filtering medium, which commonly comprises a hybrid multilayer system of melt spun polypropylene (PP) non-wovens. The system is arranged in a so-called SMS-structure, with an upper and lower layer of spun bond and a middle layer of melt blown serving as the main filtering medium [4]. In addition to a necessary low fiber diameter of around 2–4 μm, the melt blown layer is characterized by a corona discharge to adhere droplets/particles electrostatically [3–11]. Both features control the filtration efficiency of the FFP2 mask [4]. On the one hand, the fiber diameter defines the pore size of the non-woven network and therefore is required to be as small as possible to hinder the diffusion of particles/droplets in the relevant size scale in a mechanical way. On the other hand, an electrostatic charge (so-called electret) of the melt blown layer enables an adhesion of particles/droplets, which is essential to ensure the desired filtering of particles/droplets with a size smaller than the pore size of the non-woven and thus the respective filtering standard [3,4,11].

Environmental factors that result from normal-use-applications or strategies to decontaminate used masks can influence the functionality of the electret and are therefore important to be investigated [5–10]. An exposure to moisture and/or elevated temperatures is unlikely to reduce the filtration efficiency significantly [12–19] with a retained value of around 95% at a particle size of 0.3 μm as reported by several previous studies [15–17,19]. It can be supposed that the characteristic electret layer of the melt blown masks is still intact after treatment. Nevertheless, there is a study reporting a significant drop in dipole charge density of some electret masks after a heat treatment for up to 90 minutes at 70°C and 150°C, respectively, but retaining a filtration efficiency above 90%. It was concluded that if the non-woven structure and the resulting pores of the main filtering layer are dense enough, the mask could still perform sufficiently by only filtering mechanically [18]. However, this aspect strongly depends on the construction characteristics of the face mask and therefore cannot be transferred to all mask types of the same filtering standard. Reflecting the results of the previous studies presented, some aspects remain unclear due to differences in the experimental design as well as uncertainties and irregularities regarding the investigated particle/droplet size range. Therefore, in the present study we performed a comprehensive analysis of the impact of different environmental conditions (i.e. variation of temperature and relative humidity (RH) of the air as well as more aggressive measures such as autoclave sterilization or exposure to a discharging IPA atmosphere) on the filtration behaviour of various FFP2 masks. Variation of temperature and RH together with autoclave sterilization are linked to physical sterilization methods while exposure to IPA describes a chemical one. A resistance against sterilization would allow to reuse contaminated face masks and would therefore help to reduce the ecological challenges resulting from the massively increased demand of face masks [1,8]. Several previous studies suggested the application of certain physical sterilization methods like autoclaving [17,20–23] or UV light decontamination [15,23–25] for being less critical. Focusing on autoclave sterilization, it is expected not to result in a significant reduction of filtration efficiency below 95% at a particle size of 0.3–0.5 μm as a previous study has shown investigating N95 certified mask types [17]. On the other hand, the use of gamma radiation for physical sterilization was described to be rather critical for the filtration efficiency with reduced values around 70% [26,27]. However, chemical treatment with alcohols like ethanol [15,23] or IPA [22,23] are expected to diminish the filtration ability considerably due to a significant discharge of the electrostatically treated melt blown layer down to only a few percent of its original dipole charging value [18,22]. However, one study has reported that filtration capabilities retained sufficient after IPA treatment with a filtration efficiency drop of only about 7–15%. The same study presented similar results after treatment with vaporized hydrogen peroxide (VHP) [18], which is another chemical normally known to lead to critical impairment of the filtration efficiencies of respirators as it is also the case with bleach or ethylene oxide (EO) [15,22,23,27].

In addition to conventional electrostatically treated melt blown FFP2 masks, we investigated also newly available nanofibrous FFP2 polymer masks with no electret but only a mechanical principle of filtering. Masks of this type are unlikely to be affected by treatments that reduce the filtration efficiency by damaging the electret and the electrostatic bonding forces. However, their layer structure is more complex, typically including a nanoporous membrane as it was the case with the nano-masks investigated in terms of this study [28,29]. Therefore, marginal physical defects could already impair their filtration efficiency. Another example for nano-masks are masks consisting of an electrospun nanofibrous filtering layer. The potential of these kind of masks was summarized in a review study pointing out the opportunities to introduce a transparent face mask [30]. The research on novel nanofibrous type masks is generally of big scientific interest as it promises enhanced opportunities, especially in filtration efficiency, as some recent studies have shown [30–33]. Therefore, the present study also focuses on the question whether these novel but partly more expensive masks have advantages in terms of their overall filtration performance after exposure to different environmental conditions. First, the fiber diameters of all composing layers of all mask types were determined to get an idea for the mechanical filtration ability. Secondly, the filtration efficiency was measured together with the pressure drop as a quality factor before and after a certain treatment.

## Materials and methods

We purchased six different FFP2 mask models (M1–M6) from different suppliers. Table 1 shows the mask models with the corresponding price for an order of around 50 masks per type. The designation in the brackets represents the corresponding one further used in the context of this study.

**Table 1:** Mask models M1–M6 with the corresponding price.

Sample name	Mask model, supplier	Price (€/piece^a^)
M1	Simplecase, Shangdong Shengquan New Materials Co., Ltd., Jinan, China **(Simplecase)**	0.43
M2^b^	Air Queen Breeze, Siegmund Care, Oberottmarshausen, Germany (**Siegmund Care)**	1.17
M3	Aura– 9320D+, 3M, St. Paul, U.S.A. (**3M Aura**)	2.01
M4	D/Maske–Modell 2, exbert GmbH & Co. KG, Unna, Germany (**D/Maske**)	1.48
M5	TF-9006, Wellwhizz Tengfei Technology Co., Kunshan City, China (**Wellwhizz**)	1.00
M6^b^	Nano Mask, Casada Deutschland GmbH, Paderborn, Germany (**Casada Nano**)	5.95

^a^ on July 2021.

^b^ nanofibrous mask with no electret.

Table 2 lists the available information provided by the supplier on the structural design of mask models M1, M2, M4 and M6. No specific statements for models M3 and M5 were found with the exception that M3 has a special 3-piece design with PP as filter material [34] and M5 has a 5-fold filter system consisting of nonwoven, spun bond and melt blown [35]. It is worth mentioning that the nanofibrous mask types M2 and M6 have an atypical structure. They comprise special nanoporous membranes consisting of polyethylene terephthalate (PET) with added polyvinylidene fluoride (PVDF) (M2) and polytetrafluoroethylene (PTFE) (M6), respectively, instead of a PP melt blown layer typically used for filtration.

**Table 2:** Overview of the available manufacturer’s specifications for the structure of the masks.

Layer^a^	M1 [36]	M2 [28]	M4 [37]	M6 [29]
**1**	Molding layer: PP nonwoven	PP spun bond	Protective layer	Cotton functional fabric with antibacterial and antiviral finishing agent
**2**	Filter: PP melt blown	Filter: 98.4% PET + 1.6% PVDF	Special filter layer	Composite filter layer: non-woven fiber layer + nano PTFE protective membrane
**3**	Bacteriostatic layer: biomass graphene PP nonwoven	PP spun bond	Special filter layer	
**4**	Skin-friendly layer: PP nonwoven	−	Skin-friendly inner nonwoven	Cotton functional fabric with antibacterial and antiviral finishing agent

^a^ from outside to inside.

Table 3 provides an overview on the performed treatments together with corresponding treatment parameters.

**Table 3:** Overview of the performed treatments (K1, K2 and K3) and treatment parameters ^1-3^.

Treatment	Description	Implementation
K0	Control group	No treatment or aging (use as received after ordering)
K1^1^	Altered temperature and humidity	Step 1: 24 h, 100°C → step 2: 48 h, 55°C, 95% RH → step 3: 24 h, -40°C
K2^2^	Electret discharge by IPA treatment	24 h exposure in saturated IPA atmosphere
K3^3^	Sterilization by autoclaving	20 min, 121°C, 2 bar

^1^ conducted after standard VW LAH 000.819 by *Fiatec–Filter- und Aerosoltechnologie GmbH*.

^2^ conducted in a Topas discharge chamber TDC 584 after standard ISO 16890–4 by *Fiatec–Filter- und Aerosoltechnologie GmbH*.

^3^ conducted in a Selectomat PL (MMM Münchener Medizin Mechanik GmbH, Germany) after internal standards by the Central Processing Unit for Medical Devices, University Hospital Erlangen.

**Fig 1:**
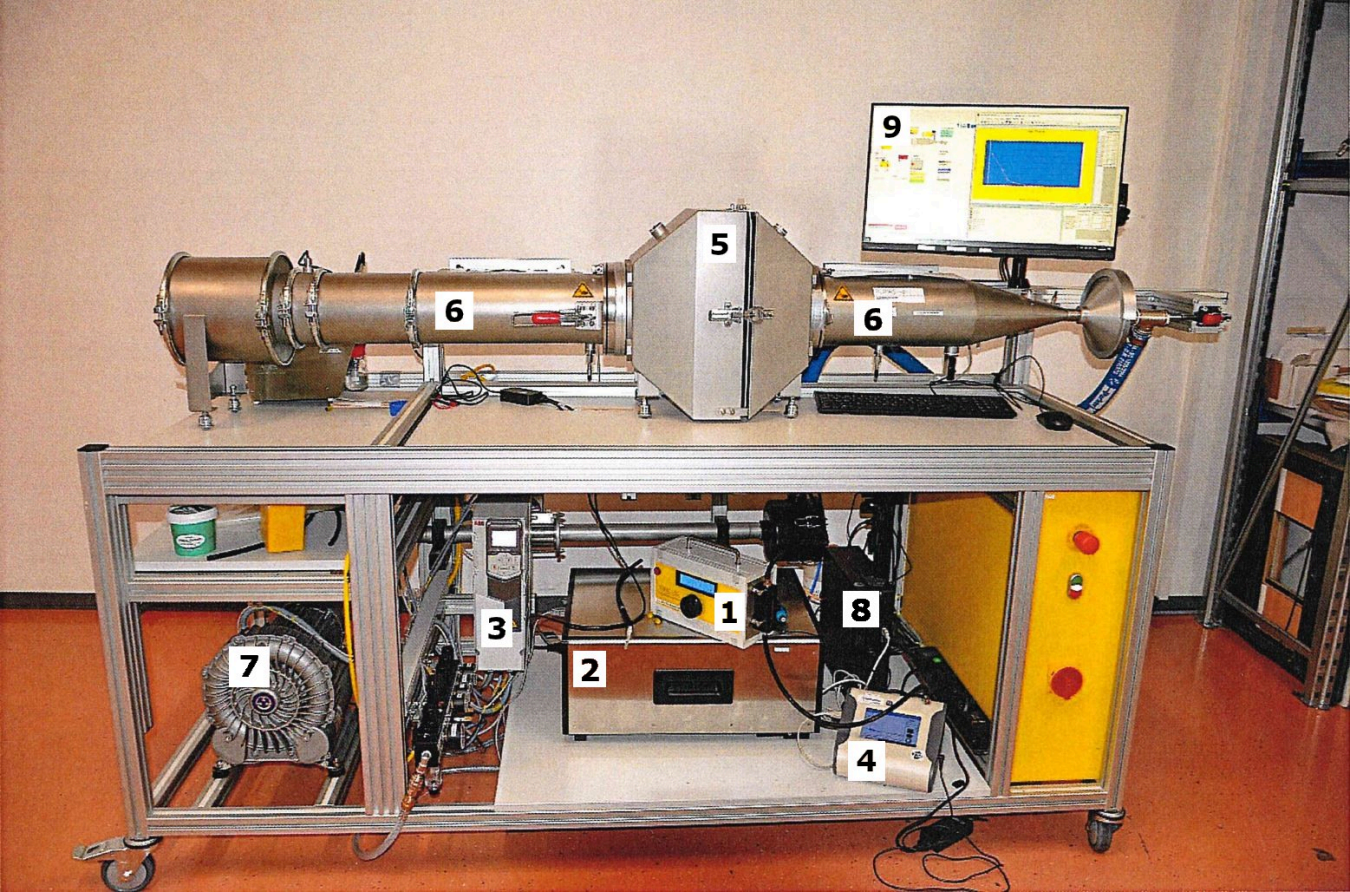
Experimental setup of filtration efficiency and pressure drop measurement.

The company *Fiatec–Filter und Aerosoltechnologie* also performed the measurements of filtration efficiency after the standard CWA 17553, which consisted of the fractional collection efficiency and the pressure drop. The experimental setup is shown in Fig 1 with the related designation shown in Table 4. The related testing conditions comprised a volume flow of 95 L/min with a face velocity of 8 cm/s, temperature of 23°C ± 1°C, RH of 50% ± 3% and particle range of 0.1–7.5 μm. Three particle sizes representing the different filter categories or mimicking a viral pathogen were applied: 0.6 μm (FFP2), 0.3 μm (N95), 0.1 μm (SARS-CoV-2) [4]. The particles were counted with a Laser Aerosol Spectrometer 3340A (TSI Inc., Shoreview, U.S.A.). The testing aerosol was paraffin oil genereated with an ATM 222 (Topas GmbH, Dresden, Germany) and without an elecrostatic neutralization. Two samples of each mask model were tested, while the second mask sample served for validation of the results from the first mask sample tested. The presented filtration efficiencies represent the average particle collections with corresponding standard deviations calculated from three sets of up- and downstream measurements of the first mask sample.

**Table 4:** Designation of the experimental setup from Fig 1.

Number	Designation
1	Paraffin oil generator ATM 222 (Topas GmbH, Germany)
2	Laser Aerosol Spectrometer 3340A (TSI Inc., U.S.A.)–implemented
3	General Scanning Mobility Particle Sizer (SMPS) 3938 (TSI Inc., U.S.A.)–not implemented
4	Optical Particle Sizer (OPS) 3330 (TSI Inc., U.S.A.)–not implemented
5	Testing chamber with mask sample and connection to flow and pressure sensor
6	Aerosol flow chamber
7	Pressure pump
8	Computer
9	Monitor

By combining the filtration efficiency, represented by the fractional collection efficiency, *η* and pressure drop Δ*p*, a quality factor *QF* can be defined according to Eq 1 [38,39] The quality factor reflects the filtration properties as well as the protective effect and comfort of breathing for humans and therefore describes the overall quality of a FFP2 mask.


$$QF=\frac{\ln\left(\frac{1}{1-\eta }\right)}{\Delta p}$$ (1)

The masks were optically analysed by scanning electron microscopy (SEM) (*CrossBeam*, Carl Zeiss AG, Oberkochen, Germany) to determine the corresponding fiber diameter of all composing layers. At least 50 fibers were measured per layer for statistical analysis with the software *ImageJ*.

## Results and discussion

### Fiber diameter

**Fig 2:**
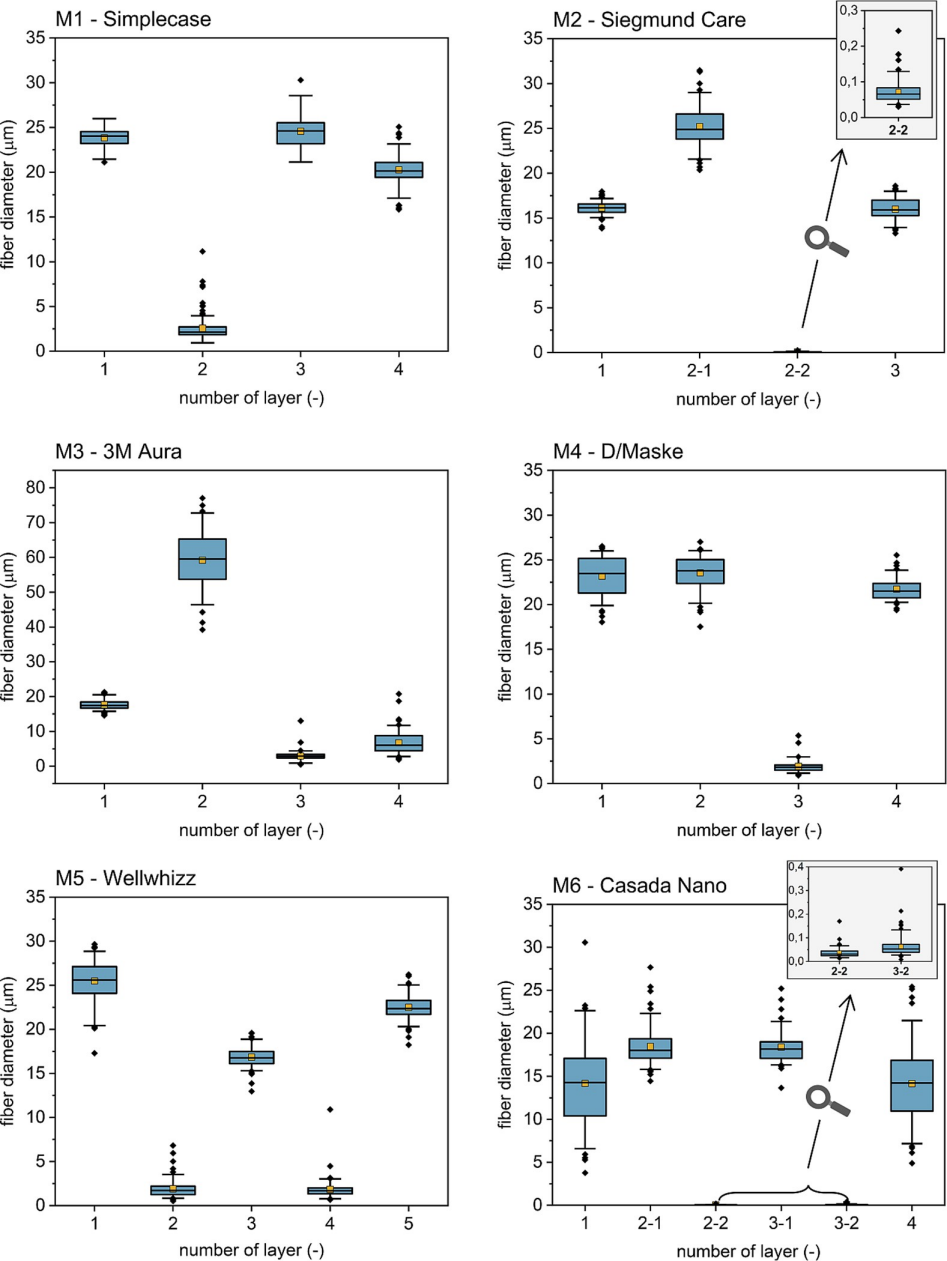
Fiber diameter distribution of all composing layers for each mask model (M1–M6).

The distributions of the fiber diameters of all composing layers are depicted in Fig 2 for each mask model.

The boxplots show the mask structure as well as the fiber diameter distribution with mean and median. The box is restricted by the lower and upper quartiles. Thus, the middle 50% of the fiber diameters are inside the box. The line in the box indicates the median and the yellow square the mean. Whiskers are limited to 5% and 95% of the fiber diameter. Outliers characterize anything beyond this. The masks have a different number of layers, ranging from three to five. Here, layer 1 indicates the outer protective layer and the highest number indicates the inner nonwoven, which is in contact with the skin. The additions -1 and -2 for layers 2 and 3 of M2 and M6 stand for the analysis of both sides of the layer, since a nano-layer with a fiber diameter in a range of around 20–200 nm is applied to one side.

Measurements of the fiber diameters revealed that the four tested masks, which have at least one inner melt blown layer (M1 –layer 2, M3 –layer 3, M4 –layer 3 and M5 –layer 2 and layer 4, showed a median fiber diameter of 2 μm. In contrast, the median diameter of the nanofibers was in the range of 30–65 nm, which is around 50 times smaller than the fiber diameter of the melt blown layers. The outer layers, which are often spun bond fabrics and are used to support the melt blown fabrics, were found to have a larger fiber diameter of 15–30 μm. The additional support layer 2 in M3 consisted of fibers with diameters in the range of 40 μm to 80 μm. However, layer 2 is only present in the mouth-part of the 3-part mask M3, which provides additional support and stability, whereas the nose and chin area of M3 consists of only three layers and thereby remains flexible.

### Filtration properties

**Fig 3:**
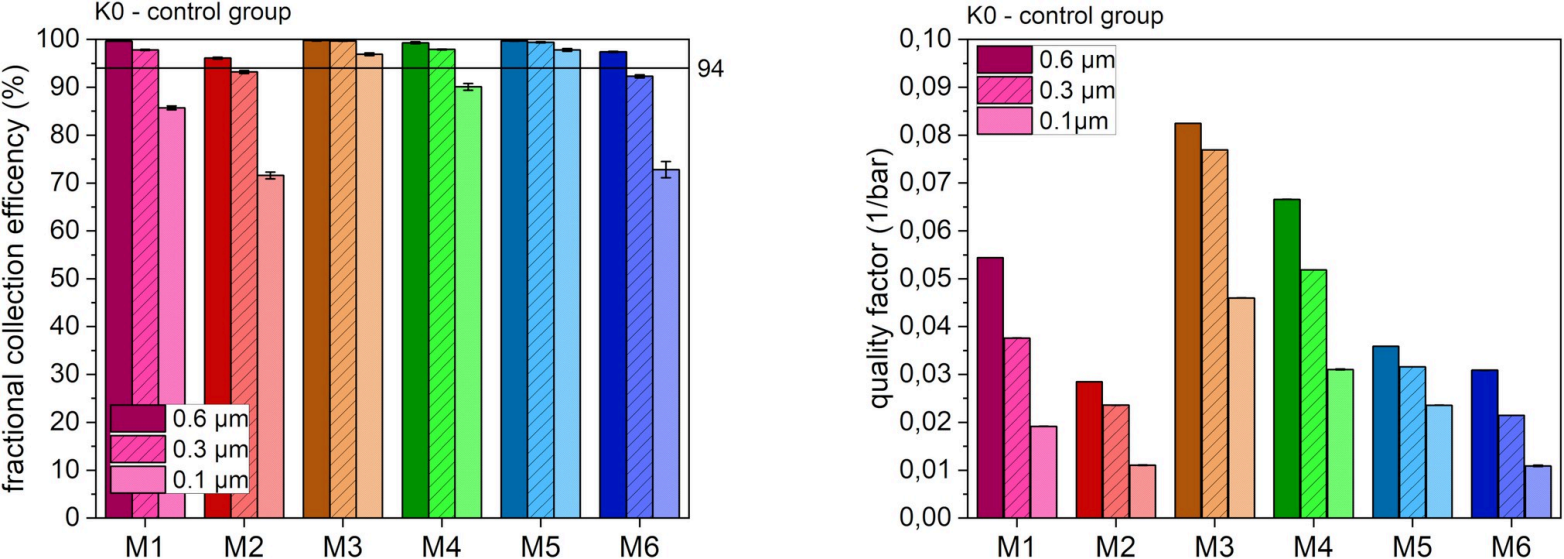
Fractional collection efficiency (left) and quality factor (right) after K0 at different particle sizes.

For all six types of masks, the filtration properties were calculated from the fractional collection efficiency and the pressure drop, leading to the quality factor (Eq 1). Fig 3 summarizes the fractional collection efficiency and quality factor for all mask types without prior treatment (i.e. control condition K0).

In the absence of any treatment, all mask models fulfilled the FFP2 standard. The best filtration efficiencies were observed with the electret melt blown masks (M1, M3, M4, and M5), which showed values above 99%. Even when the smallest particle size of 0.1 μm was used for testing, three masks (M3, M4 and M5) still exhibited a filtration efficiency of 90% and higher. In contrast, the nano-masks (M2, M6) showed an insufficient filtration efficiency of only around 70% at this particle size. With respect to the quality factor, the electret melt blown mask models M3 and M4 were clearly superior to the nanofibrous models M2 and M6, with a roughly four times higher value in case of M3 for all particle sizes. This indicates that although the nanofiber masks consists of fibers with the smallest diameter (Fig 2), they do not exhibit the highest filtration efficiency. Instead, the electrostatically treated melt blown masks with median fiber diameters that were up to a magnitude higher showed the best filtration capacity, which illustrates the significant impact of charging on filtration properties. Although in general these differences do not seem to be crucial as the FFP2 standards were still fulfilled by all masks, they might become relevant if the filtration of particles below 0.6 μm is needed.

**Fig 4:**
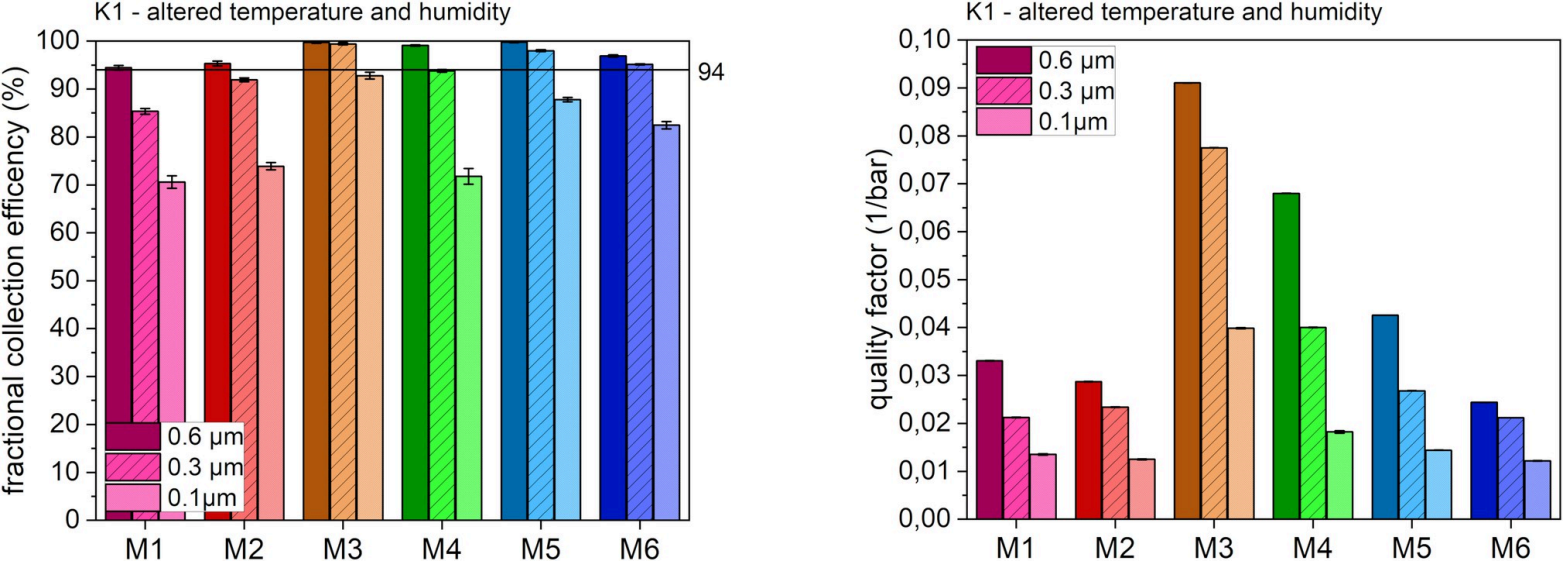
Fractional collection efficiency (left) and quality factor (right) after K1 at different particle sizes.

Fig 4 shows the filtration properties after the sequential exposure of the masks to different temperatures and humidities (condition K1).

Changes between high and low temperatures and humidity had no pronounced effect on the filtration performance of all mask models. For the various mask models, the same trends in differences between the masks were observed as under control conditions (K0, Fig 3). Five of six masks still fulfilled the FFP2 standard, except for M1 (where the fractional collection efficiency was 0.5% below the cut-off after K1 treatment). M1 also turned out to be the only mask model with a significant decrease of the quality factor by approximately 50% for all particle sizes (compare Fig 4, right panel) with Fig 3, right panel).

**Fig 5:**
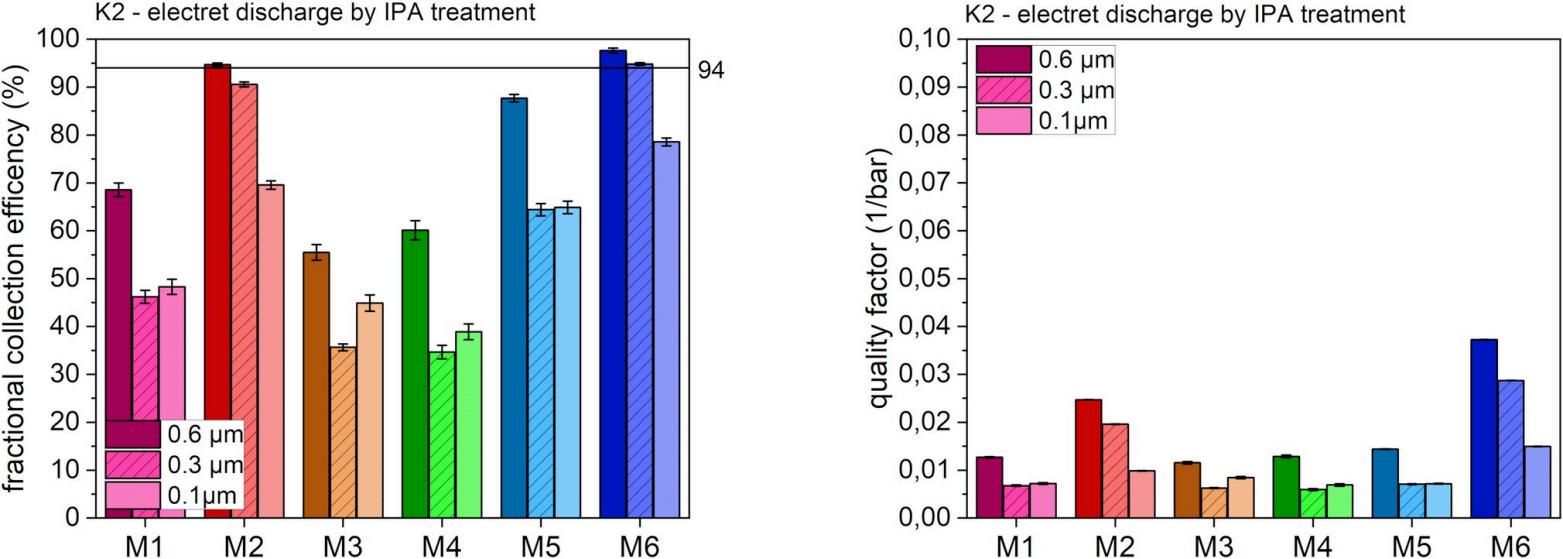
Fractional collection efficiency (left) and quality factor (right) after K2 at different particle sizes.

The results after exposure of the masks to IPA (treatment condition K2) are depicted in Fig 5.

As expected, electrostatic discharging by IPA had the greatest impact on the filtration efficiency and quality factor of the electret melt blown masks. All four masks (M1, M3, M4 and M5) failed to match the FFP2 standards after treatment with IPA. For example, the filtration efficiency for model M3 dropped to 50%. At particle sizes below 0.6 μm, only values of around 30% were reached. In the case of model M5, the decrease of filtration performance was somewhat less pronounced, but the filtration efficiency was still insufficient. For all electret melt blown models, the quality factor fell to around 15–30% of the original K0 value. The nano-masks (M2 and M6), on the other hand, showed no significant decrease regarding filtration efficiency and quality factor due to the absence of electrostatic treatment. These results underline the importance of an intact electret on the melt blown layer to enable sufficient protection. Our results are therefore only partly in accordance with the findings of a study mentioning that IPA treatment had only a marginal effect on the filtration efficiency [18]. If the mechanical ability of filtering is sufficient enough like it was the case with the nano masks, no negative effect of IPA is to be expected. On the other hand, if filtration capabilities are mainly characterized by electrostatic charging, IPA treatment is to be considered as critical.

**Fig 6:**
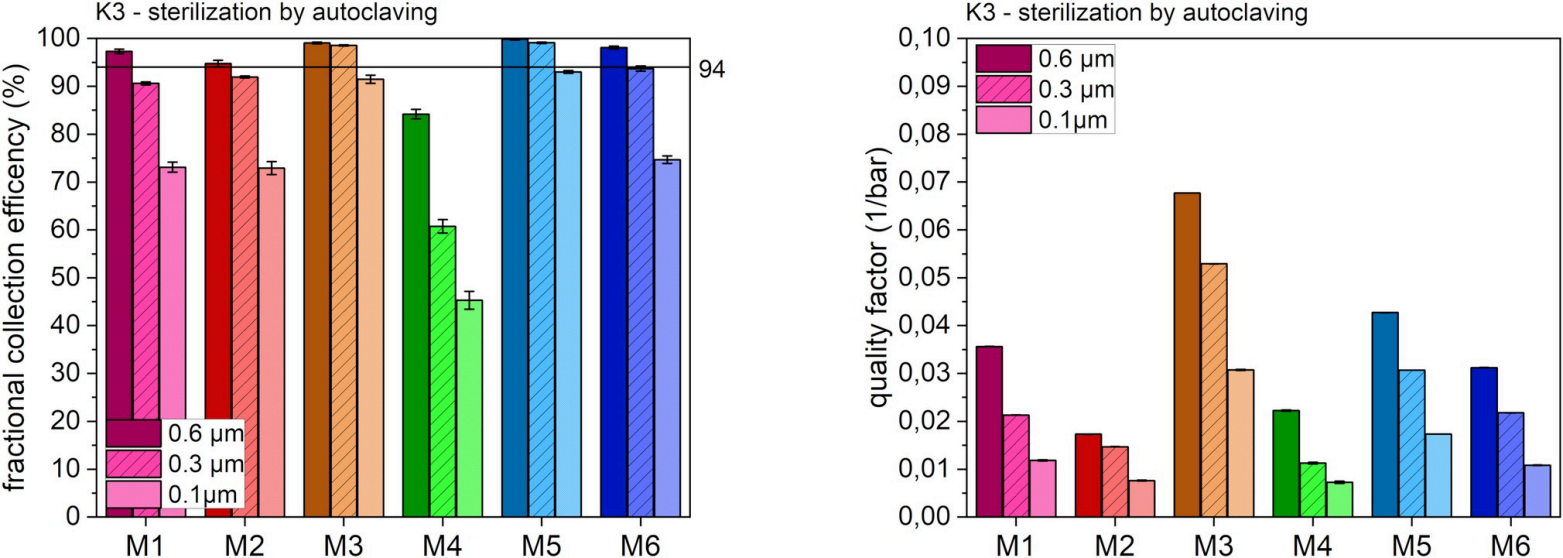
Fractional collection efficiency (left) and quality factor (right) after K3 at different particle sizes.

In Fig 6, the results of sterilization of masks by autoclaving (treatment condition K3) are presented.

Autoclaving had no relevant impact on the filtration efficiency and quality factor for five of the six mask models. The overall trends in differences were similar to those seen under the treatment condition K1. In the case of mask model M4, autoclaving caused a remarkable decrease of the filtration efficiency of around 15% and 30% for a particle size of 0.6 μm or 0.3 μm, respectively. In addition, a major loss in the quality factor was observed with a reduction of around two thirds for all particle sizes. It is assumed that unlike to the other melt blown masks (M1, M3 and M5) the electret treatment of model M4 suffers by autoclaving, which leads to a worse filtration ability.

## Conclusion

In this research, we investigated the influence of certain environmental factors on the filtration performance of six different FFP2 mask models with a focus on three different particle sizes (0.6 μm, 0.3 μm, 0.1 μm) that represent certain filtering stages. Four of the analysed face mask models contained a common layer of an electrostatically treated PP melt blown nonwoven as the main filtering medium, whereas two face mask models had an uncharged nanofibrous layer where the filtration is solely based on mechanical retention of the particles. The fiber diameters of the filtering medium were in the range of around 2–4 μm for the electret melt blown and between 20 and 200 nm for the nano-masks, respectively.

Taken our results together, it is fair to state that across a broad particle range down to 0.1 μm the conventional FFP2 masks with an electret melt blown layer as a filtering medium showed a better overall filtration performance and breathability than the novel nanofibrous masks. Environmental conditions (K1) and even autoclaving (K3) were not able to impair the filtering capacity significantly leading to new possibilities in terms of reusability. Only an extreme form of exposure as it was the case with discharging by IPA (K2) incapacitated all electret FFP2 masks as expected. Although the nano-masks showed resistance against such an artificial exposure, their overall filtration capacity was clearly inferior to electret FFP2 masks under normal use application. Considering the results obtained with the nano-mask M6 (“Casada Nano Mask”), its high price of nearly 6 €/piece can hardly be advocated. The electret melt blown mask M3 (“3M Aura”) achieved by far the overall best filtration results which somehow justifies the elevated price of around 2 €/piece. However, even inexpensive masks with a price of around 1 €/piece showed a mostly sufficient performance. Finally, it is important to emphasize that the protective effect of a FFP2 mask not only depends on its filtration capacity, but also on its proper wearing with no leakages to prevent an unfiltered airflow. Furthermore, a possible deterioration of the face mask layer material has to be considered for further reusability after applying certain treatment conditions like the ones covered in the scope of this study.

## Supporting information

S1 File (DOCX)

S2 File (DOCX)

S3 File (XLSX)
