# Supplementary material for: Influence of different treatment conditions on the filtration performance of conventional electret melt blown non-woven and novel nano FFP2 masks
Source: PLoS One. 2023 Sep 21;18(9):e0291679. doi: 10.1371/journal.pone.0291679 (PMC10513275; doi:10.1371/journal.pone.0291679)
Supplement: S1 File — (DOCX) [file pone.0291679.s001.docx]

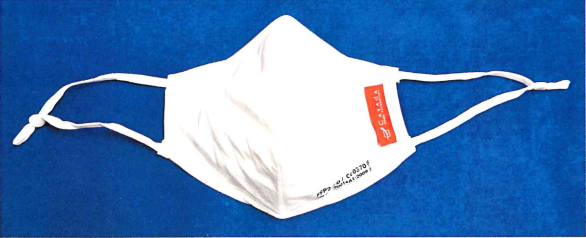

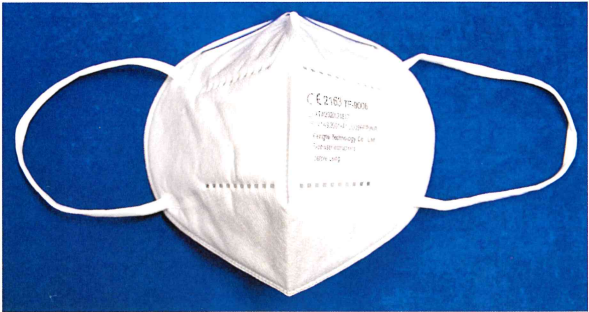

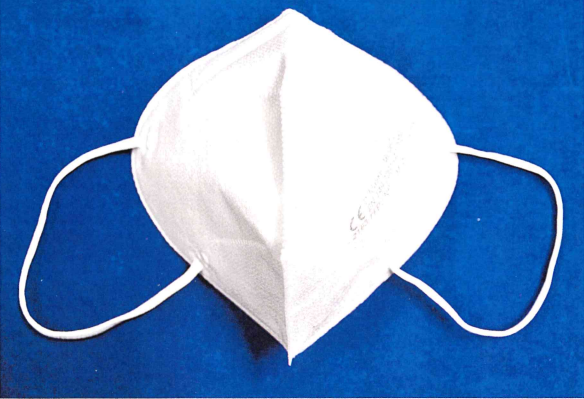

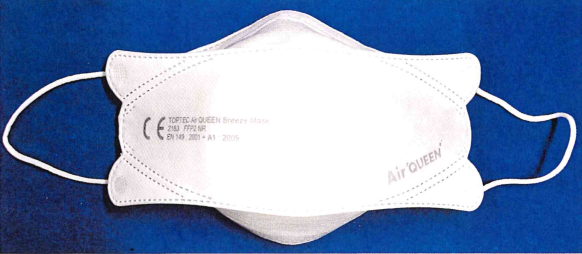

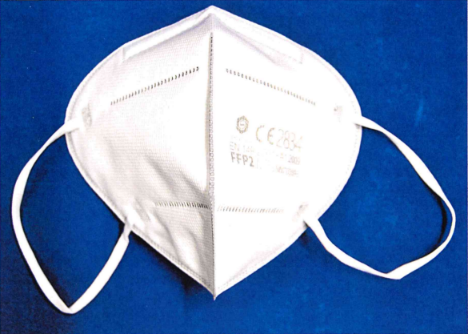

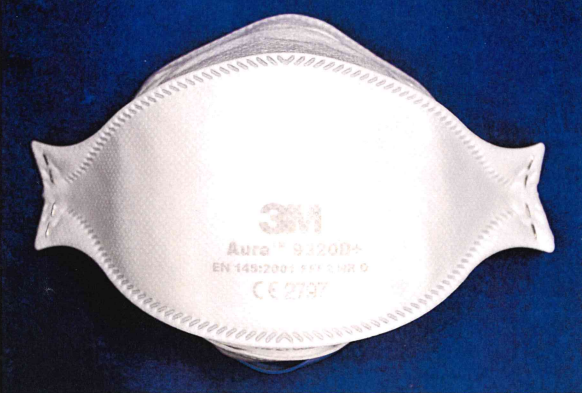


M2 – Siegmund Care

**S1 Fig: Overview of mask models M1-M6 at condition K0 (untreated, as received)**

M4 – D/Maske

M6 – Casada Nano

M5 – Whellwhizz

M3 – 3M Aura

M1 – Simpecase
