# Supplementary material for: Influence of different treatment conditions on the filtration performance of conventional electret melt blown non-woven and novel nano FFP2 masks
Source: PLoS One. 2023 Sep 21;18(9):e0291679. doi: 10.1371/journal.pone.0291679 (PMC10513275; doi:10.1371/journal.pone.0291679)
Supplement: S2 File — (DOCX) [file pone.0291679.s002.docx]

**S2 Tab: Pressure drop for all mask models M1-M6 after condition K0-K3**

|  |  | Pressure drop (bar) for conditions K0-K3 | | | |
| --- | --- | --- | --- | --- | --- |
| Sample Name | Mask model | K0 | K1 | K2 | K3 |
| M1 | Simplecase | 101,5 | 90,5 | 91,5 | 111 |
| M2 | Siegmund Care | 114 | 107,5 | 120,5 | 171,5 |
| M3 | 3M Aura | 75,5 | 66 | 70,5 | 80 |
| M4 | D/Maske | 74,5 | 69,5 | 71,5 | 83 |
| M5 | Wellwhizz | 162 | 146 | 146 | 153,5 |
| M6 | Casada Nano | 119,5 | 143 | 103 | 127 |
